# Dataset on part replacement of dipalmitoylphophatidylcholine with locust bean on stimulated tracheobronchial fluid, *in vitro* bioaccessibility test and modeling of lung deposition of trace elements bound to airborne particulates

**DOI:** 10.1016/j.dib.2019.105010

**Published:** 2019-12-26

**Authors:** Emmanuel Gbenga Olumayede, Ilemobayo Oguntimehin, Chekwube C. Ojiodu, Bolanle M. Babalola, Ayomipo Ojo, Olagboye S. Adeoye, Olubunmi G. Sodipe

**Affiliations:** a Department of Industrial Chemistry, Federal University, Oye, Ekiti, Nigeria; b Department of Chemical Sciences, Ondo State University of Science and Technology, Okitipupa, Ondo State, Nigeria; c Department of Science Laboratory, Yaba College of Technology, Lagos Nigeria; d Department of Industrial Chemistry, Ekiti State University, Ado, Ekiti, Nigeria; e Department of Animal Environment and Biology, Federal University, Oye, Ekiti, Nigeria

**Keywords:** Tracheobronchial fluid, Bioaccessibility test, Particulates, Trace elements, Lung deposition

## Abstract

The data presented in this article are related to our work on development of tracheobronchial fluid, *in vitro* bioaccessibility test and modeling of lung deposition of trace elements bound to airborne particulates [1]. In this article, a neutral modeled tracheobronchial fluid was formulated by partial replacement of some constituents in recipes of previously used lung epithelium fluids with local materials and was used in *in vitro* bioaccessibility extraction of elements-bound to airborne particulates. Dataset of particulate matters-bound trace elements collected in selected locations Ado – Ekiti is presented and the deposition of elements in different regions of respiratory tracts is estimated using Multiple-path particle deposition (MPPD) mathematic model. The data reveals that the formulated fluid has physical characteristics with superior properties to the existing fluids. The data also shows that bioaccessibility of elements were generally low (<30%) except for Cd and As with relatively moderate values (between 45 and 50%). Additionally, the lung deposition modeling shows that greater percentage of Cd (about 40% of inhaled dose) deposition in the lower alveolar part of the respiratory tract while tracheobronchial and extra-thoracic had 33% and 27% respectively. The data sets can be used as references to analyze data obtained using other formulation.

**Table:** Specifications Table

Subject	*Toxicology*
Specific subject area	*Air pollutant and Toxicology*
Type of data	*Table and Figure*
How data were acquired	*Sampling method: Active sampling of PM 2.5 using Dichtomous Sampler (Model 241, Rupprecnt and Patashrick manufactured), and Software model: MPPD version 2.1 software was downloaded* via http://www.ara.com/products/mppd.htm*. to estimate the regional particles deposition in respiratory tract and retention in the respiratory tract*
Data format	*Raw and graph*
Parameters for data collection	*tracheobronchial fluid used for this work was formulated using recipe partially substituted with locus bean, bioaccessibility of particulate matters-bound trace elements were analyzed*
Description of data collection	*Formulation of tracheobronchial fluid, bioaccessibility, exposure dose and lung deposition*
Data source location	*Ado -Ekiti, Ekiti State, Nigeria* (*Longitude 5.150*^*0*^*E, Latitude 7.100*^*0*^*N)*
Data accessibility	*The raw data files are archived in FUOYE Repository*http://repository.fuoye.edu.ng/handle/123456789/2251 *Data is available from the corresponding author upon reasonable request.*
Related research article	*E.G Olumayede* et al. *“Development of tracheobronchial fluid, in vitro bioaccessibility assessment of particulates-bound trace elements. MethodsX 6 (2019) 1944–1949* [1]

**Table:** 

**Value of the Data** • Data in this article format can be explored in risks assessment of exposure to inhale particulate matters in the area • The data can be used for inter-laboratory comparison of bioaccessibility of element-bound particulates • The data generated could stimulate environmental concerns on the impacts of airborne particulates • The data could be translated into improved respiratory health among the people exposed to ambient particulate matters.

## 1 Data

**Fig. 1:**
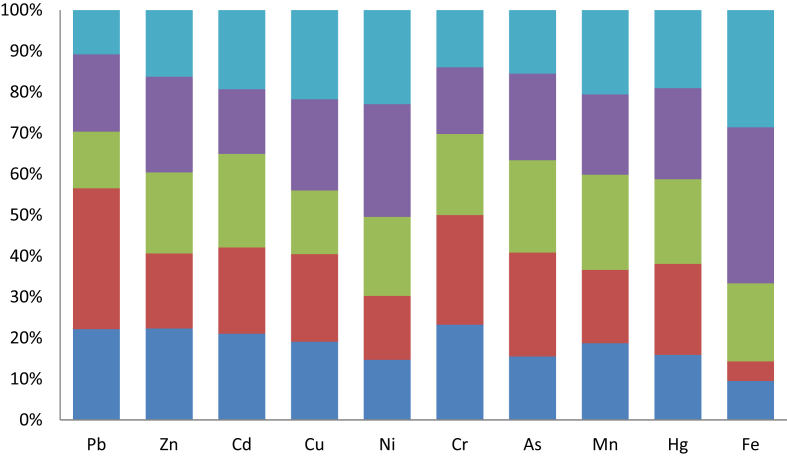
Bioaccessibility (%) of the various metals at different sites of the data collection locations using tracheobronchial airway fluid. Navy Blue, red, green, purple and light blue represent Fajuyi, Oja-Oba, Secretariat, Ilokun and Poly Road sites respectively.

**Fig. 2:**
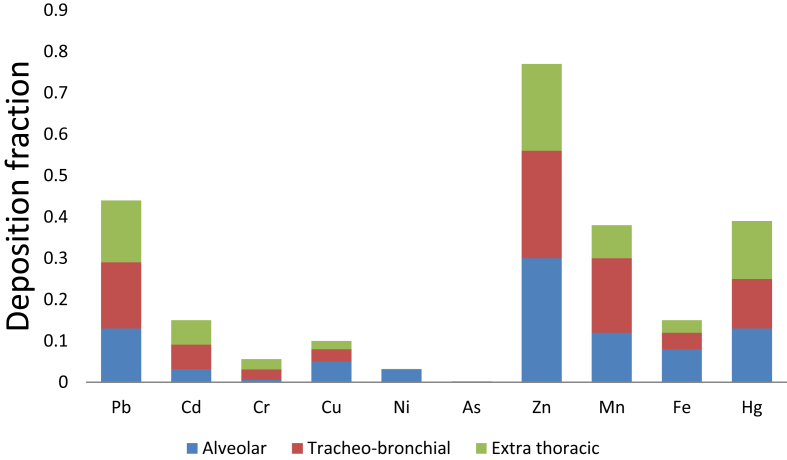
Deposition fractions of various metals in regions of human respiratory tract. Blue, Red and Green bars represent deposited fractions in the respiratory tract.

The dataset in this article describes the bioaccessibility of elements-bound airborne particulates collected in selected locations of Ado – Ekiti, an urban center in Southwest, Nigeria using modeled tracheobronchial fluid. Table 1 describes the recepies of the modeled tracheobronchial fluid in which locally avaliable materials as substituent. Fig. 1 describes bioaccessibility *in vitro* extraction test of the various metals at different sites of the data collection locations using the formulation. Fig. 2 displays the estimated regional particles deposition in respiratory tract using MPPD version 2.1 software.

**Table 1:** Recipe for synthetic tracheobronchial fluid.

	Reagent	Concentration (mg/L)
Inorganic	NaCl	6020
	Cacl_2_.2H_2_O	256
	Na_2_HPO_4_	150
	NaHCO_3_	2700
	KCl	298
	MgCl_2_	200
	Na_2_SO_4_	72
Surfactant	DPPC	100
Thickening agent	Locus beans gum	350
Chelating agent	Citrate	50
Large-Molecular mass Protein	Albumin	260
Cross-linking agent	polyethylene oxide resins	500
Antioxidant	Ascorbic acid	18
Organic acids	Glycine	376
	Cysteine	122

DPPC means dipalmitoylphophatidylcholine.

## 2 Experimental design, materials, and methods

### 2.1 Formulation and preparation of tracheobronchial fluid

The recipe of formulated fluid used for this work was described in previous works [1,2], where Gambles’ solution was modified [3,4] and used in *in vitro* extractions and to evaluate bioaccessibility [[5], [6], [7], [8], [9], [10], [11]] of some elements in healthy non-smoking human. The compositions of the fluid contain Inorganic and organic constituents mixed in ultra-pure water. The modifications to the fluid in this work include the use of locus beans gum, as thickening agent and addition of weak chelating agent (citrate) and antioxidant (Ascorbic acid). Furthermore, polyethylene oxide resin was used to replace mucin, as crosslinking agent, due to their availability.

### 2.2 Description of data collection center

The data collection center for this article is Ado – Ekiti, the administrative headquarter of Ekiti state, southwest Nigeria and lies on Longitude 5.150^0^E, Latitude 7.100^0^N. It is a fast growing urban settlement that has witnessed tremendous growth resulting from rural-urban migration into the city since it was named as the capital city of the Ekit state in 1996. According to population census of 2006 report, Ado - Ekiti has about 484,798 inhabitants [12], and by now should be close to 1 million. The city is impacted by particulate matters from anthropogenic activities; such as traffic, open burning of waste, aerosols from unpaved roads and construction works.

### 2.3 Sampling of airborne particulates matters over Ado - Ekiti

Samples of airborne particulates were collected in five (5) selected sites of Ado - Ekiti city using a Dichtomous Sampler (Model 241, Rupprecnt and Patashrick manufactured) at 2-week intervals for 5 months from November 2016 to March 2017 at height of 1.5–2 m. Five sampling sites were carefully chosen within the city, to reflect different human activities impacting the environments. The sampler consists of an omnidirectional aerosol inlet and a virtual impactor assembly along with tripod stand. It collects particles in two size ranges; d < 2.5 μm and 2.5 < d < 10μm, which are collected on two separate PTFE Teflon (37mm diameter) filters. The sampler operates at a flow rate of 16.7Lmin^−1^for 2 hours. For each sampling, the filters were equilibrated in a charged desiccator for 24 hours and weighed before and after sampling. After weighing, particulates were stored in a refrigerator at 4 °C until analysis. A total of 15 samples were collected in each site during sampling period of this work. The samples were then prepared for elemental analysis and bioaccessibility test.

### 2.4 Bioaccessible fraction of elements bound to particulate matters

Bioaccessibile of elements bound to airborne particulates collected at different locations were determined in *in vitro* extraction test. For the purpose of this dataset, we used PM_2.5_ particle size only. Samples were placed into five different a 15-ml polypropylene centrifuge tube, with 0.25g of particulates per tube. Each sample was mixed with 10 ml of formulated tracheobronchial fluid, and the tube was made airtight and immersed for 2 h in a shaking water bath at 37 °C (1-h shaking followed by 1 h still). The obtained extractants were cooled to room temperature, then centrifuged for 20 min at 3500 rpm and separated for element determination. The bioaccessibility (%) was determined as the soluble fraction of an element in PM that dissolved in tracheobronchial fluid [9].

### 2.5 Modeling exposure and lung deposited doses

The amount of airborne particulates intake via inhalation exposure pathway is computed as described in previous article [13]. Multiple-path particle deposition (MPPD) mathematical model [14,15] was employed to estimate deposition of elements in different regionals of the lung (alveolar, trachea-bronchial and extra thoracic) in children and adults. The MPPD version 2.1 software was downloaded via http://www.ara.com/products/mppd.htm. The human airway morphometric model selected for this dataset was the Yeh/Schum symmetric. Other parameters chosen for the dataset include inhalant properties (aerosol), Breathing scenario (endotracheal); time (1), mass concentration (1 mgm^−3^), Breathing frequency (120 1/min), and tidal volume (625 ml). The data input includes size of the particulate (Count medium diameter, CMD) [16].

(1) $$Inhalability=1-{\left[1+e^{\left(13.56+0.4343\right)}X\;\log\;dae\;-4.88)\right]}^{-1}$$

where, d_ae_ (μm) is the aerodynamic particle diameter.
