# Supplementary material for: Dataset on part replacement of dipalmitoylphophatidylcholine with locust bean on stimulated tracheobronchial fluid, in vitro bioaccessibility test and modeling of lung deposition of trace elements bound to airborne particulates
Source: Data Brief. 2019 Dec 26;28:105010. doi: 10.1016/j.dib.2019.105010 (PMC7096670; doi:10.1016/j.dib.2019.105010)
Supplement: Multimedia component 1 [file mmc1.docx]

**Supplementary data**

**Estimation of exposure to airborne particulates**

The amount of airborne particulates intake via inhalation exposure pathway is computed from the equation

$$Inhalation intake= Airborne particulate concentration \times Breathing rate \times Exposure time\ldots\ldots\ldots\ldots\ldots\ldots\ldots\ldots\ldots\ldots\ldots\ldots\ldots(1) .$$

The contribution from individual metal to inhalation dose is calculated as follows:

$ID(\mu g/day)= \frac{Cmetal \times TR\times∁pm \times Vresp}{BW} \times100$………………………………………..… (2)

Where, DF= deposited fraction of particle in different regions of respiratory tract, C = concentration of metal inhaled per volume of air (particle cm^-3^), t = the exposure time (hours), IR. = Lung rate, BW = body weight

The individual doses are summed to yield a total inhalation rate using equation

Inhalation dose = $\sum intakej\times Di$ …………………………………………… (3)
